# Successful Complete Laparoscopic Resection of Benign Peritoneal Multicystic Mesothelioma Adherent to the Ascending Colon: A Case Report

**DOI:** 10.70352/scrj.cr.25-0271

**Published:** 2025-09-25

**Authors:** Yoshihito Kitamura, Masashi Miguchi, Ryo Nagao, Makoto Shinohara, Keigo Nakashima, Michinori Hamaoka, Masakazu Hashimoto, Toshihiro Misumi, Nobuaki Fujikuni, Satoshi Ikeda, Yasuhiro Matsugu, Keiichi Mori, Takashi Nishisaka, Hideki Nakahara

**Affiliations:** 1 Department of Gastroenterological Surgery, Hiroshima Prefectural Hospital, Hiroshima, Hiroshima, Japan; 2 Department of Pathology and Laboratory Medicine, Hiroshima Prefectural Hospital, Hiroshima, Hiroshima, Japan

**Keywords:** benign peritoneal multicystic mesothelioma, colon, laparoscopic surgery

## Abstract

**INTRODUCTION:**

Benign peritoneal multicystic mesothelioma (BPMM) is a rare mesothelial tumor with a high local recurrence rate and potential for malignant transformation. It predominantly affects women of reproductive age and is often associated with prior abdominal surgery or inflammation. Complete surgical resection is the standard treatment; however, tumor recurrence remains a concern. BPMM occurring outside the pelvis is extremely rare, and lesions requiring colectomy because of adherence to the gastrointestinal tract are infrequent.

**CASE PRESENTATION:**

A 63-year-old woman presented with right lower abdominal pain. She had undergone cervical cancer surgery 20 years ago. Contrast-enhanced CT and MRI revealed multiple well-defined cystic lesions adjacent to the ascending colon. Colonoscopy revealed extrinsic compression, and ^18^F-fluorodeoxyglucose (^18^F-FDG)-PET/CT showed no abnormal uptake of ^18^F-FDG. Given the patient’s symptoms, a laparoscopic right hemicolectomy with intracorporeal anastomosis was performed. The cystic lesions were firmly adhered to the appendix, cecum, and ascending colon, requiring *en bloc* resection. Histopathological and immunohistochemical analyses confirmed a diagnosis of BPMM. The patient had an uneventful postoperative course and was discharged on POD 7. No recurrence was observed during the 1-year follow-up period.

**CONCLUSIONS:**

A laparoscopic approach may be a feasible and safe option for complete resection of BPMM as it allows for magnified visualization and careful handling of cystic lesions, avoiding their intraoperative rupture. Given the high recurrence rate of BPMM, close postoperative surveillance is essential. This case illustrates the feasibility of laparoscopic resection for BPMM.

## Abbreviations

BPMM
: benign peritoneal multicystic mesothelioma
CD
: cluster of differentiation
CK
: cytokeratin
CRS
: cytoreduction surgery
^18^F-FDG
: ^18^F-fluorodeoxyglucose
HIPEC
: hyperthermic intraperitoneal chemotherapy

## INTRODUCTION

Benign peritoneal multicystic mesothelioma (BPMM) is a rare primary tumor arising from the mesothelial layer of the visceral peritoneum.^1)^ The characteristic behavior of BPMM is benign; however, potentially malignant transformation has been reported.^2)^ BPMM often occurs in women of reproductive age, develops in the pelvis, and is associated with previous abdominal surgery, endometriosis, or pelvic inflammatory disease.^3)^ Pathological examination is essential to diagnose BPMM, and surgical tumor resection is commonly performed for diagnosis and treatment.^4,5)^ Herein, we report the case of a patient with a multicystic mass observed adjacent to the ascending colon on CT and confirmed as BPMM on pathological examination. This case report is expected to enrich the existing literature further and highlight the key points regarding surgical resection for BPMM adherent to the gastrointestinal tract.

## CASE PRESENTATION

**Fig. 1:**
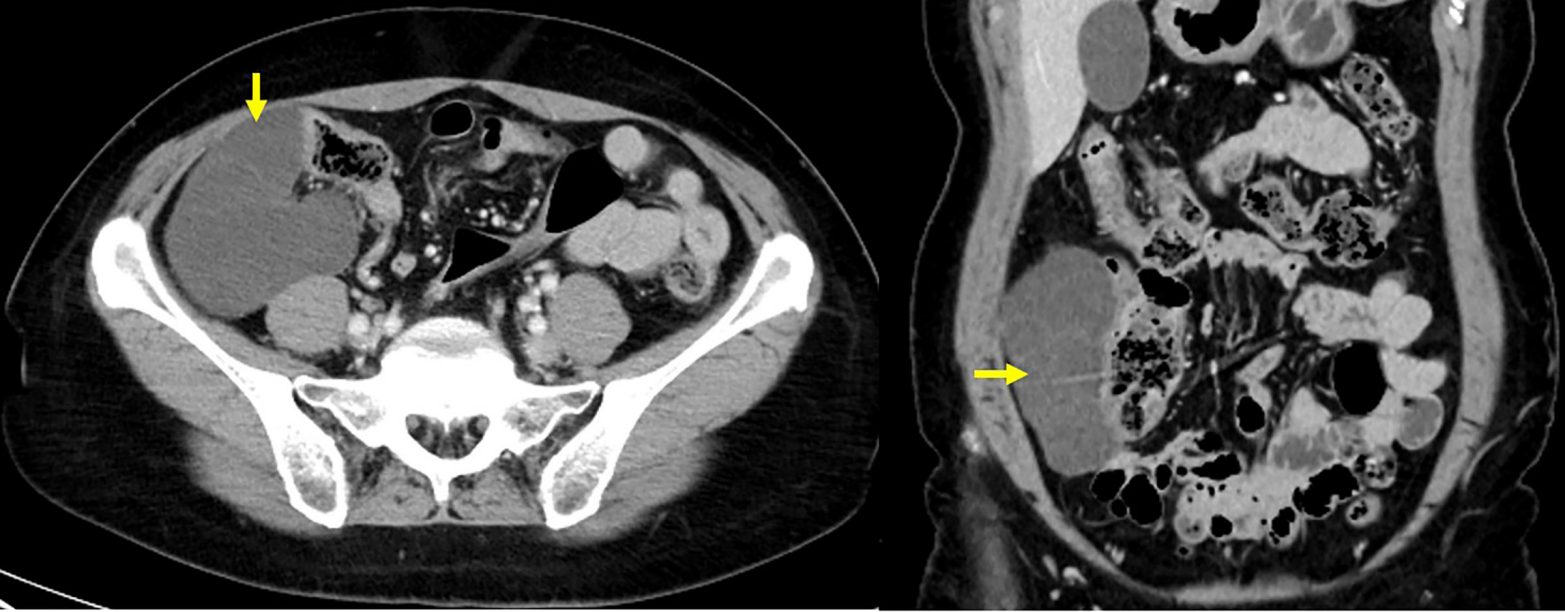
Contrast-enhanced CT findings. The CT images show multicystic lesions located adjacent to the ascending colon. The yellow arrow indicates the tumor.

**Fig. 2:**
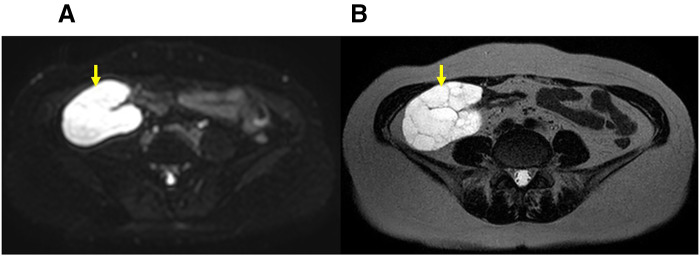
MRI findings. (**A**) Diffusion-weighted image of the tumor. (**B**) T2-weighted image of the tumor. MRIs show multiple cystic lesions with clear boundaries, numerous septa, and no solid components. A clear boundary is observed between the lesions and the ascending colon. The diffusion-weighted image shows homogeneous, high signal intensity within the tumor. The yellow arrow indicates the tumor.

**Fig. 3:**
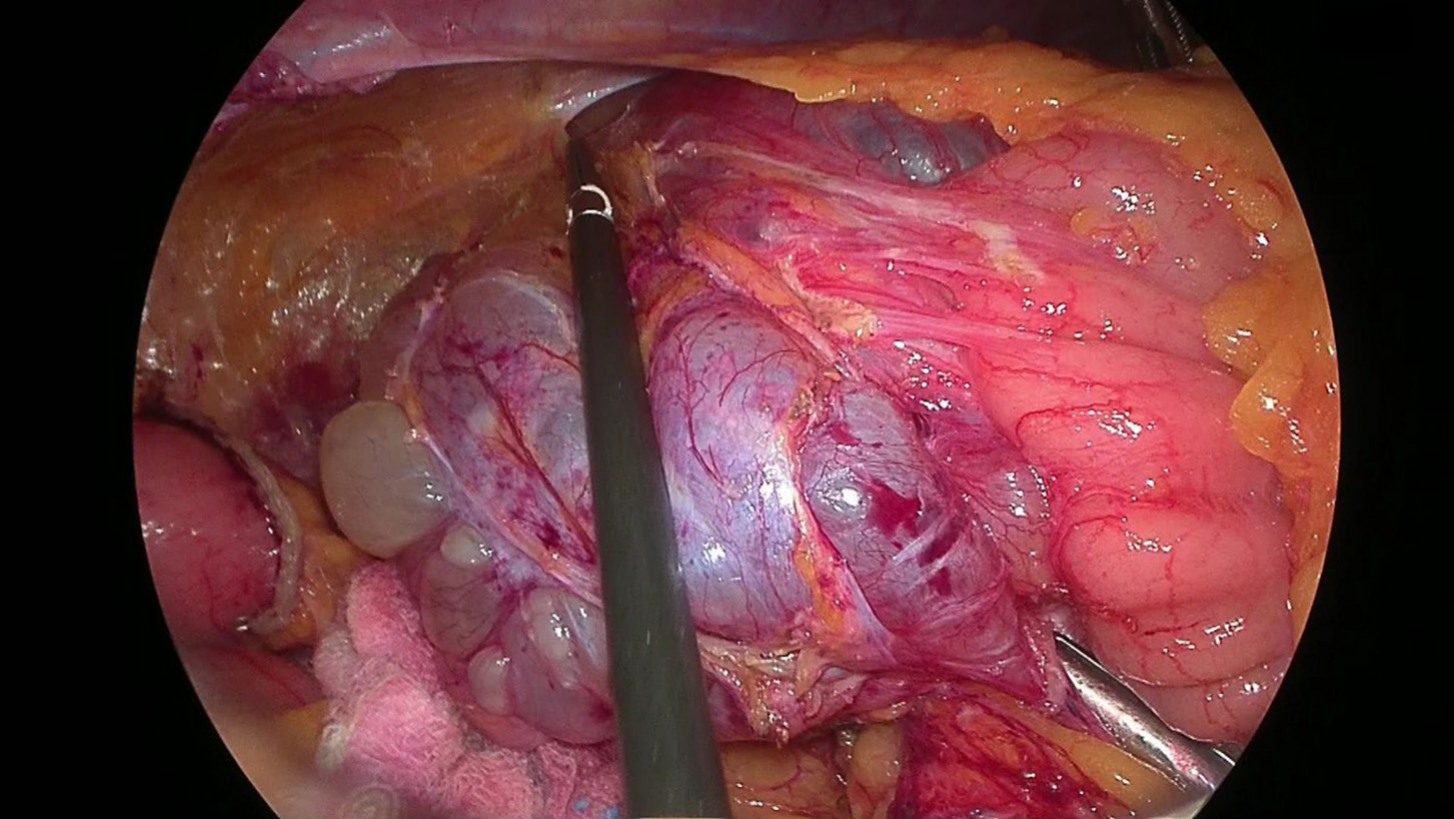
Intraoperative findings. Multicystic lesions adherent to the appendix, cecum, and ascending colon are seen.

**Fig. 4:**
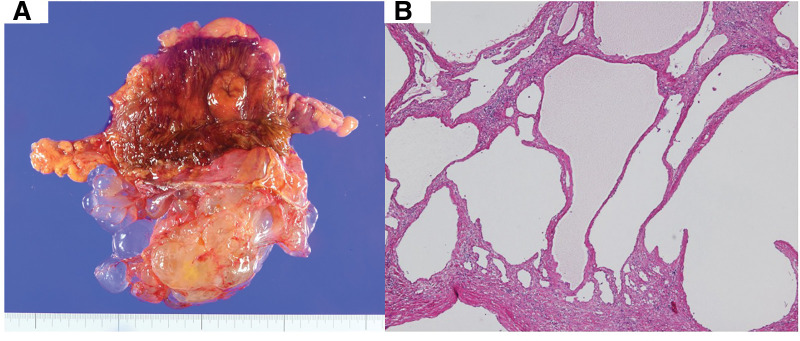
The resected tumor tissue. (**A**) Macroscopic findings. (**B**) Hematoxylin and eosin staining of the tumor tissue (magnification ×40).

**Fig. 5:**
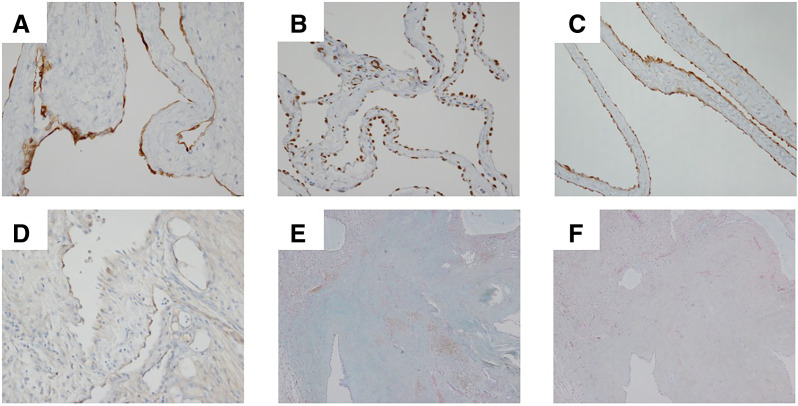
Immunohistochemical staining of the tumor. The tumor tissue shows positive staining for CK5/6 (**A**), Wilm’s tumor-1 (**B**), calretinin (**C**), and D2-40 (**D**) (magnification ×200). The tumor tissue shows weak positive staining for alcian blue (**E**) and negative staining for hyaluronidase alcian blue (**F**) (magnification ×40).

A 63-year-old woman was admitted to our surgical department with the chief complaint of lower right abdominal pain. The patient had undergone surgery for cervical cancer 20 years ago. Physical examination revealed a soft, slightly tender mass in the right lower abdomen. Laboratory test results did not reveal any abnormalities. Contrast-enhanced CT revealed multiple nonspecific cystic lesions with a maximum diameter of 113 mm located adjacent to the ascending colon. No enhancement was noted after intravenous administration of contrast agents (**Fig. 1**). Colonoscopy revealed extrinsic compression of the ascending colon. MRI also revealed multiple cystic lesions with clear boundaries, numerous septa, and no solid components. The boundary between the lesions and the ascending colon was clear, and no findings suggestive of invasion into the colon were observed (**Fig. 2**). ^18^F-fluorodeoxyglucose (^18^F-FDG)-PET/CT showed no increase in ^18^F-FDG uptake by these lesions or other organs. Based on these findings, the differential diagnoses included abdominal lymphangioma, enteric duplication cysts, and multicystic peritoneal mesothelioma. Because the patient presented with abdominal pain, surgery was performed for diagnosis and treatment. The patient underwent laparoscopic right hemicolectomy with an intracorporeal delta-shaped anastomosis. Intraoperatively, multicystic lesions adhering to the appendix, cecum, and ascending colon were observed (**Fig. 3**). Because of the difficulty in detaching them, the lesions and right-sided colon were resected *en bloc*. Histologically, the lesions consisted of cysts lined with a single layer of flattened, polygonal, or cuboidal cells without atypia (**Fig. 4**). Immunostaining of the tumor tissue revealed positive results for cytokeratin (CK) AE1/AE3, CK5/6, Wilm’s tumor-1, and calretinin; weak D2-40 and Alcian blue stain positivity; local positivity for cluster of differentiation (CD) 31; and negative results for CD34, Factor VIII, and hyaluronidase alcian blue stain (**Fig. 5**). The final diagnosis was BPMM. The postoperative course was uneventful, and the patient was discharged on POD 7. One year after the surgery, no signs of recurrence were observed on CT.

## DISCUSSION

BPMM is a particularly rare disease, with fewer than 200 cases reported.^4)^ Chronic inflammation, previous abdominal surgeries, endometriosis, and recurrent peritonitis associated with peritoneal dialysis are considered as the predisposing factors of BPMM.^4–7)^ BPMM is most common in women under 30 years of age. Therefore, the present case of BPMM in a 60-year-old woman is extremely rare.^7,8)^ The period between the primary surgery and the development of this tumor is reported to range from 6 months to 20 years.^7,9)^ In the present case, the patient had no history of inflammatory disease such as appendicitis. Her previous surgery for cervical cancer might have caused BPMM development. The multicystic lesions were located adjacent to the ascending colon; however, the border between the intestine and the tumor was well-defined, with no findings indicating that the tumor originated from the intestinal wall. Therefore, it was suspected that the tumor originated from the peritoneum. Differential diagnoses of BPMM include cystic lymphangioma, malignant peritoneal mesothelioma, endometriosis, and pseudomyxoma peritonei.^4,7)^ Immunohistochemical examination is essential for the diagnosis. In lymphangiomas, endothelial markers such as CD34, CD31, Factor VIII, and VEGFR3 are typically positive.^7)^ In the present case, cuboidal cells were observed, suggesting a tumor of mesothelial origin. Immunohistochemical staining was positive for most mesothelial markers (CK5/6, calretinin, Wilm’s tumor-1, and D2-40), and Alcian blue staining was negative after hyaluronidase treatment, suggesting the presence of hyaluronic acid in the tumor. These findings confirmed the diagnosis of BPMM, a tumor of mesothelial origin. In the present case, BPMM was located adjacent to the right colon and required *en bloc* resection of the colon. BPMM is generally known as a pelvic tumor, and unlike that observed in the present case, a location outside the pelvis is rare.^4,7,10)^ To the best of our knowledge, few previous cases of this tumor arising in the right colon have been reported, in which tumor removal combined with colon resection was required.^10,11)^ Randomized controlled trials have reported that laparoscopic approaches have equivalent short- and long-term outcomes compared with open surgery for colon cancer.^12,13)^ Furthermore, laparoscopic surgery for a colon cancer lesion >8 cm in diameter has short-term feasibility, achieving a rapid return of bowel function and a short length of stay compared with open surgery.^14)^ In the present case, a colectomy combined with cyst removal was expected to involve challenges similar to those encountered in cases of colon cancer with a tumor diameter of >8 cm. However, considering the expected superior short-term outcomes, laparoscopic surgery was deemed a safe and feasible option. The advantages of laparoscopic surgery include identifying small cystic lesions owing to the magnification effect, which allows complete resection of the tumor, including these lesions. However, laparoscopic surgery involves linear forceps manipulation; therefore, cystic lesions should be gently handled, and intraoperative rupture of these lesions should be carefully attended to. Postoperative recurrence due to intraperitoneal fluid leakage following the rupture of a cystic BPMM lesion has not been reported; however, cyst rupture may result in a portion of the cyst wall remaining in the abdominal cavity, leading to incomplete tumor resection. In the present case, as the first strategy to achieve complete resection, ileal dissection was performed intracorporeally after dissecting the blood vessels to the colon using a medial approach. This facilitated exposure of the lateral cecum and ascending colon, where the cystic lesion was located, enabling dissecting around the cystic lesion without rupturing the cyst wall. As a 2nd strategy, *en bloc* resection of the adjacent organs was performed without dissection where the cystic component of the BPMM was adherent to the adjacent organs, even if radiological findings indicated clear boundaries between the cystic component and the colon. The standard treatment for BPMM is complete tumor resection; however, the recurrence rate after resection is approximately 50% after 3–27 months (mean, 32 months).^15)^ Therefore, hyperthermic intraperitoneal chemotherapy (HIPEC) is an additional treatment provided after tumor resection. HIPEC involves the combined use of cisplatin and doxorubicin.^4,7)^ The survival period may be extended by combining surgery with HIPEC. HIPEC was not performed in the present case because a definitive diagnosis of BPMM had not been confirmed at the time of surgery, and the criteria for adding HIPEC intraoperatively had not been established. It is difficult to obtain a definitive diagnosis of BPMM based on preoperative imaging tests. While some patients undergo fine-needle aspiration (FNA) before surgery, a negative cytology result reduces the possibility of a malignant tumor. However, a definitive diagnosis remains challenging, as FNA often yields nonspecific findings.^8,16–18)^ Immunohistochemical examination of the tissue is a reliable method for obtaining a definitive diagnosis. There are several case reports of BPMM diagnosed using biopsy with exploratory laparoscopy and treated with the combination of cytoreductive surgery (CRS) and HIPEC, revealing multiple implants of the tumor or adhesions with various intraperitoneal organs.^19,20)^ One case report revealed that the patient was evaluated by intraoperative frozen section and underwent CRS and HIPEC before immunohistochemical evaluation of the tumor tissue.^21)^ There are also case reports in which CRS and HIPEC were initially planned and were performed after laparoscopic evaluation. However, to date, no evidence supporting the efficacy of administering HIPEC as an adjuvant therapy following the postoperative diagnosis of BPMM exists. Risk factors predicting the recurrence of BPMM remain unknown. In the present case, although the patient has been recurrence-free for one year after surgery, conducting short-term surveillance for tumor recurrence is essential.

## CONCLUSIONS

For complete resection of BPMM adherent to the colon, the tumor and adherent colon should be resected *en bloc*. A laparoscopic approach may facilitate complete resection by enabling precise identification and excision of even small cystic lesions owing to the magnification effect.
